# Report of Exosomes Isolated from a Human Uterine Leiomyoma Cell Line and Their Impact on Endometrial Vascular Endothelial Cells

**DOI:** 10.3390/ph15050577

**Published:** 2022-05-05

**Authors:** Antonia Navarro, Maria Victoria Bariani, Hang-Soo Park, Ami R. Zota, Ayman Al-Hendy

**Affiliations:** 1 Department of Obstetrics & Gynecology, University of Chicago, Chicago, IL 60637, USA; 2012navarro@gmail.com (A.N.); bariani@bsd.uchicago.edu (M.V.B.); hspark06@bsd.uchicago.edu (H.-S.P.); 2 Department of Environment Health, Milken School of Public Health, George Washington University, Washington, DC 20037, USA; azota@gwu.edu

**Keywords:** uterine leiomyoma, fibroid, myometrium, exosomes, miRNA, heavy menstrual bleeding

## Abstract

Uterine leiomyomas are the most common pelvic tumor in women of reproductive age; they cause irregular heavy menstrual bleeding leading to anemia and subsequent negative effects on quality of life. Exosomes have arisen as main players of disease progression in several illnesses, including a range of benign and malignant conditions; however, their role in leiomyomas’ pathophysiology remains unknown. We investigated the effect of exosomes derived from human uterine leiomyoma tumor cells (HULM) and human myometrial cells (UTSM) on the behavior of human endometrial microvascular endothelial cells (HEMEC). HULM- and UTSM-derived exosomes were isolated and cocultured with HEMECs. Then, cell proliferation, mRNA expression, tube formation assay, and RNA-seq were performed. Treatment of HEMEC with HULM-derived exosomes increased cell proliferation by 60% compared to control untreated cells, upregulated C-MYC and VEGFA expression levels, and increased tube formation, length, and branching (markers of angiogenesis). Profiling of miRNA revealed that 84 miRNAs were significantly downregulated and 71 were upregulated in HULM-derived exosomes compared to UTSM-derived exosomes. These findings suggest that HULM-derived exosomes might have effects on HEMEC function, containing factors that enhance endometrial proliferation and angiogenesis, which may contribute to heavy menstrual bleeding. Further research on exosomes in uterine leiomyoma may identify possible novel biomarkers for treatment.

## 1. Introduction

Leiomyomas or uterine fibroids are the most common gynecological disorder in women of reproductive age; they undergo rapid and substantial growth [1]. Clinical outcomes include irregular and heavy menstrual bleeding, which leads to severe anemia, dysmenorrhea, pelvic pressure and pain, urinary incontinence, dyspareunia, infertility, preterm labor, and early and recurrent pregnancy loss [2,3]. Leiomyomas are present in more than 70% of women and are symptomatic in approximately 30% of them; they are the most common clinical indication for hysterectomy that prematurely ends a woman’s reproductive life [4]. Risk factors for leiomyomas include age, race, obesity, hypovitaminosis D, and endogenous and exogenous hormonal factors [5,6,7]. Despite their high prevalence, no approved pharmacotherapies provide efficient treatment, and surgery remains the main treatment option [8]. Leiomyomas carry a significant personal and economic burden, with an estimated annual healthcare cost of up to $34 billion in the US [9]. 

The exact molecular mechanisms that initiate and control the development and growth of leiomyomas have not been clearly elucidated. However, several factors including extracellular matrix remodeling, hormonal imbalance, driver mutations, epigenetic alterations, DNA damage, and miRNAs, among others have been implicated in the development and progression of leiomyomas [10,11,12,13,14,15,16,17]. Factors expressed and secreted by leiomyomas could affect endometrial cell growth, function, and vessel remodeling, communicating in an autocrine and paracrine manner and contributing to reproductive complications such as heavy and irregular menses, miscarriages, and subfertility [18]. Exosomes have arisen as main contributors of disease progression in numerous illnesses, including a variety of benign and malignant conditions; however, their function in leiomyomas etiology and pathophysiology remains underinvestigated.

Exosomes, macrovesicles, and apoptotic bodies are extracellular vesicles that are produced by fusion of multivesicular bodies with the plasma membrane and are secreted by a variety of cells, biological fluids, including the uterine fluid, and the endometrium [19,20]. Exosomes are the smallest vesicle type, with a size between 30–150 nm, and are discharged by exocytosis into the extracellular space where they influence adjacent and distant cells under various conditions [19,20,21,22,23,24,25]. A lipid bilayer membrane encapsulates the exosome, which protects their cargo from degradation and allows uninterrupted entry into recipient cells. Exosomes carry functionally active molecules including nucleic acids (DNA, mRNA, miRNA, ncRNA, and lncRNA), proteins, lipids, and other particles and transmit them to recipient cells [26]. Exosomes are fundamental mediators of intercellular signaling among neighboring cells and distant tissues, modulating gene expression, angiogenesis, migration, and tissue repair in healthy and tumor cell development [27]. 

The functional properties of exosomes have been documented in copious normal physiological and pathological conditions, including normal pregnancy and several types of cancers and chronic inflammatory illnesses [23,24,25,26,27]. To the best of our knowledge, there are no previous studies addressing exosome biology and function in patients with uterine leiomyoma or in patient-derived cell lines. To address this important gap of knowledge, the objective of this study was to portray and define the functional impact of exosomes derived from human uterine leiomyoma (HULM-EXO) and healthy myometrium smooth muscle (UTSM-EXO) cell lines on cultured human endometrial microvascular endothelial cells and identify the miRNA profile in HULM-EXO compared to UTSM-EXO. We hypothesized that HULM-derived exosomes affect the uterine microenvironment and in particular the human endometrium by carrying specific uterine leiomyoma-secreted factors. The release of HULM-EXO by leiomyomas might contribute to the pathogenesis of uterine leiomyomas by creating a vicious cycle and delivering specific miRNAs that act in an autocrine and paracrine fashion in recipient cells. 

## 2. Results

### 2.1. Isolation and Depiction of Exosomes Derived from Human Uterine Leiomyoma Cell Line

**Figure 1:**
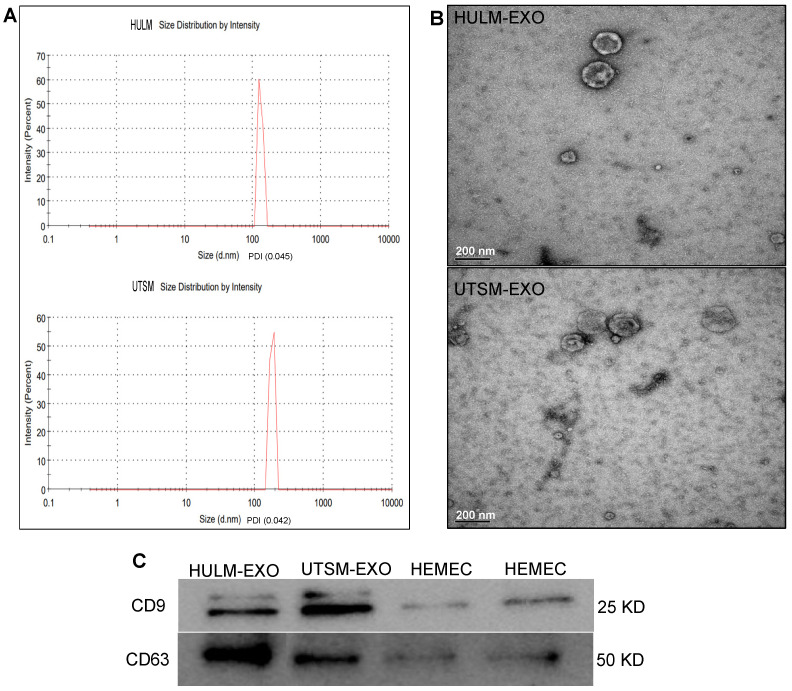
Isolation and depiction of exosomes derived from human uterine leiomyoma cell line. (**A**) nanoparticle tracking analysis (NTA) confirmed the dimension of exosomes ranging in size from 30 to 160 nm, and PDI of 0.045 and 0.042, displaying the graphs of HULM- and UTSM-derived exosomes in *X*-axis: diameter (nm) and in *Y*-axis: peak intensity. (**B**) representative micrographs of transmission electron microscopy (TEM) showed the cup-shaped expected morphology of HULM- and UTSM-derived exosomes (scale bar = 200 nm). (**C**) Western blotting demonstrated the expression of exosomal markers, CD9 and CD63, and confirmed the purity of the isolated exosomes. These images are representative of independent experiments. HULM: human uterine leiomyoma cells, UTSM: human uterine smooth muscle cells, HEMEC: human endometrial microvascular endothelial cells, and EXO: exosomes.

To investigate the physiological function of exosomes derived from HULM and UTSM cell lines on the human endometrium, we first isolated and portrayed HULM-EXO and UTSM-EXO using standard techniques. Nanoparticle tracking analysis (NTA) confirmed the expected size of HULM-EXO and UTSM-EXO (30 nm to 160 nm: Figure 1A) and the purity of the isolated exosomes, with a PDI of 0.045 for leiomyoma and 0.042 for myometrium. Transmission electron microscopy (TEM) also confirmed the presence and predictable exosomal morphology (Figure 1B). To further confirm the purity of exosomes, we performed immunoblotting for the presence of universal exosomal markers CD9 and CD63, which are tetraspanin family members (Figure 1C). We did not detect any other cellular contaminants that are normally present in cell lysates. These results confirm that HULM and UTSM cell lines release exosomes into their conditioned media, and the exosomes were successfully isolated and portrayed.

### 2.2. Uptake of Exosomes from Uterine Leiomyoma Cell Line by Human Endometrial Microvascular Endothelial Cells 

**Figure 2:**
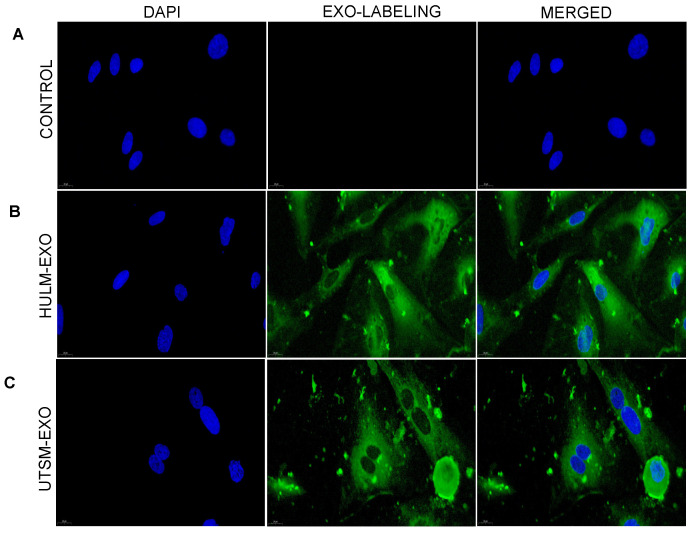
Uptake of exosomes derived from human uterine leiomyoma by human endometrial microvascular endothelial cells. (**A**–**C**) representative confocal microscopy images of labeled exosomes derived from human uterine leiomyoma (HULM-EXO) and human uterine smooth muscle (UTSM-EXO) cell lines and PBS-treated as negative control, cocultured with human endometrial microvascular endothelial cells (HEMEC) at 24 h (scale bar = 200 nm). We observed a high quantity of labeled exosomes in the cytoplasm of endothelial cells, and no exosomes were detected in endothelial cells treated with PBS, confirming the effective uptake of both HULM-EXO and UTSM-EXO on HEMEC cells.

Exosomes act in an autocrine and paracrine manner, communicating and transmitting signals to nearby and distant cells to affect their functioning. We hypothesized that HULM-EXO influence the uterine microenvironment, in particular the human endometrium, by carrying uterine leiomyoma-secreted factors. We cocultured HEMEC with fluorescently labeled purified HULM-EXO and UTSM-EXO and monitored exosome uptake via confocal microscopy. We observed HULM-EXO and UTSM-EXO uptake by human endometrial microvascular endothelial cells after 24 h of coculture (Figure 2) compared with control cells treated with PBS. This suggests that the purified exosomes can enter endothelial cells, where they could influence and alter the cell microenvironment and behavior.

### 2.3. Exosomes from Human Uterine Leiomyoma Cell Line Increase Cell Proliferation

**Figure 3:**
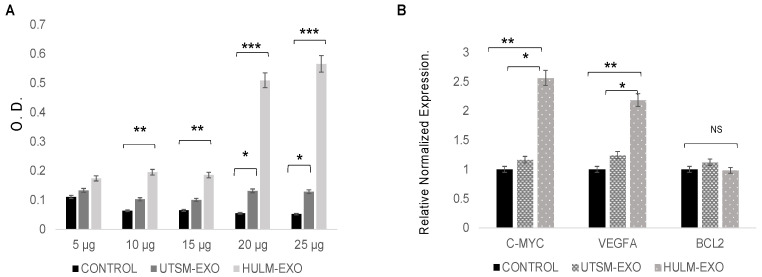
Treatment with human uterine leiomyoma-derived exosomes increases cell proliferation on HEMEC. (**A**) HEMEC cells were treated with 5, 10, 15, 20, and 25 ug/mL of HULM-EXO, UTSM-EXO, or PBS as negative control for 48 h. Then, BrdU incorporation assay was performed to measure cell proliferation. There is significant increase in cell proliferation on HEMEC cells treated at 20 and 25 ug/mL in HULM-EXO compared to UTSM-EXO or PBS treated groups. (**B**) mRNA quantification, HEMEC cells were treated with 25 ug/mL of HULM-EXO, UTSM-EXO, or PBS, followed by qRT-PCR to check for the proliferation marker: C-MYC, the angiogenic marker VEGFA and the apoptosis marker BCL2. C-MYC and VEGFA mRNA levels were upregulated on HEMEC cells treated with HULM-EXO compared to cells treated with UTSM-EXO or PBS (* *p* < 0.05), while no changed was observed in BCL2 expression. Values were statistically significant when *p* was <0.05. (** *p* < 0.01, *** *p* < 0.001), NS: not significant.

To assess the functional effect of HULM- and UTSM-derived exosomes on the endometrial endothelial cells, we examined cell proliferation of HEMEC after coculture with HULM-EXO or UTSM-EXO. BrdU incorporation assay was performed based on a time course experiment; 48 h treatment demonstrated the highest increase in cell proliferation (data not shown). The most significant growth in HEMEC proliferation was observed at 20 and 25 ug/mL HULM-EXO compared to UTSM-EXO and PBS treatments (Figure 3A). Treatment with HULM-EXO during 48 h also increased expression of the cell proliferation marker C-MYC and angiogenic marker VEGFA in HEMEC, as measured by qPCR. HULM-EXO treatment led to significant increase in mRNA levels of C-MYC and VEGFA, while there was no change in expression of the apoptosis marker BCL2 (Figure 3B). These data suggest that secreted factors in leiomyoma cell line stimulate C-MYC to drive cell proliferation and upregulate VEGFA expression, suggesting a pro-angiogenic effect in human endometrial microvascular endothelial cells.

### 2.4. Exosomes from Human Uterine Leiomyoma Cell Line Cocultured with HEMEC Demonstrate Angiogenic Properties

**Figure 4:**
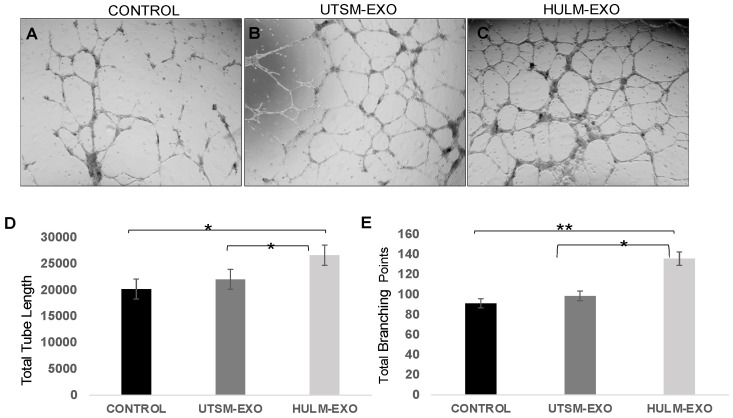
Exosomes from human uterine leiomyoma demonstrate angiogenic properties. (**A**–**C**) HEMEC cells were plated on Matrigel basement membrane matrix and treated with 30 ug/mL of HULM-EXO, UTSM-EXO, or PBS to check for changes in angiogenic properties. (**D**) HEMEC cells treated with HULM-EXO demonstrated greater tube formation and higher total tube length compared with those treated with UTSM-EXO or PBS as negative control (* *p* < 0.05). (**E**) Significant increase in total branching points was observed on HEMEC cells treated with HULM-EXO compared with those treated with UTSM-EXO and PBS (* *p* < 0.05). Values were statistically significant when *p* was < 0.05. (** *p* < 0.01).

To further examine the effects of exosomes derived from leiomyoma cell line on the physiology of the endometrium, we cocultured HEMEC with HULM-EXO, UTSM-EXO, or PBS and monitored angiogenic markers (tube formation, length, and branching). HEMEC cells plated on Matrigel basement membrane matrix treated with HULM-EXO demonstrated greater tube formation (Figure 4A–C) and approximately 25% greater total tube length compared with cells treated with UTSM-EXO (*p* < 0.05) or PBS (negative control; Figure 4D). Nearly 40% increase in total branching points was also observed in HEMEC treated with HULM-EXO compared with those treated with UTSM-EXO (*p* < 0.05) or PBS (Figure 4E). The greater tube formation, total tube length, and total branching points observed in HEMEC treated with HULM-EXO suggest that leiomyoma-derived exosomes, but not exosomes derived from healthy uterine tissue, exhibit pro-angiogenic properties, which might contribute to the abnormal menstrual bleeding observed in women with leiomyomas. 

### 2.5. MicroRNA Profiling of HULM- and UTSM-Derived Exosomes

**Figure 5:**
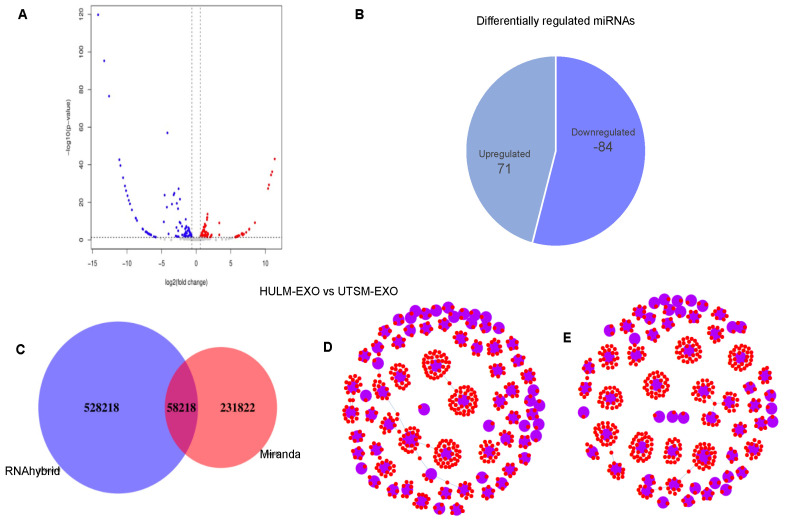
MicroRNA profiling of exosomes from HULM and UTSM cell lines. (**A**) A volcano plot demonstrated a differential pattern of miRNA expression between HULM-EXO and UTSM-EXO sample. The gray dots represent the miRNAs without differential expression, the blue dots represent the differentially downregulated miRNAs, and the red dots represent the upregulated miRNAs. (**B**) Statistical analyses demonstrated that there were 71 differentially upregulated and 84 downregulated miRNAs in HULM-EXO compared to UTSM-EXO (*p* < 0.05). (**C**) The target genes of known and novel miRNAs were predicted using miRanda and RNAhybrid software, and the corresponding relationship between miRNAs and target genes were obtained; Venn diagram showed the miRNA prediction target genes. (**D**,**E**) Network relationships of differentially downregulated and upregulated miRNA candidate target genes were performed, the blue dots indicated miRNA (known and novel), the red dots indicated the candidate target gene, and the lines indicated the interaction.

Previous studies have demonstrated that exosomes can carry genetic material between cells, including DNA, RNA, and miRNA [19,20,21,22,23,24,25,26,27]. To investigate whether exosomes from HULM and UTSM cell lines exhibit different miRNA profiles, we compared their small RNA content using an NGS platform. Analysis of all mapped reads revealed a high proportion of rRNA and tRNA in both HULM-EXO and UTSM-EXO, which is consistent with previous data characterizing exosome miRNA profiles [28,29,30]. Sample correlation analysis between HULM-EXO and UTSM-EXO showed a low correlation coefficient, indicating differential expression of miRNAs, and possibly in their miRNA cargos. We performed cluster analysis of differentially expressed miRNAs, which showed clustering of differentially expressed miRNAs in HULM-EXO compared to UTSM-EXO samples. Differences in miRNA expression in HULM-EXO versus UTSM-EXO samples were also visualized by volcano plot (Figure 5A). Statistical analysis identified 84 differentially downregulated miRNAs (49 with known function and 34 are unknown mRNAs) and 71 upregulated miRNAs (48 with known function and 23 are unknown mRNAs) in HULM-EXO compared to UTSM-EXO (*p* < 0.05; Figure 5B). In the Supplementary Materials, Table S1 showed the list of downregulated miRNAs and Table S2 showed the list of upregulated miRNA in HULM-EXO compared to UTSM-EXO. Next, the target genes of known and novel differentially expressed miRNAs were predicted using miRanda and RNA hybrid software. miRanda predicted 528,218 target genes and RNAhybrid predicted 231,822 target genes; 58,218 miRNA target genes were identified by both platforms (Figure 5C). We also performed a network analysis of the differentially downregulated and upregulated miRNA target genes (Figure 5D, E). The blue dots indicate miRNA (known and novel), the red dots indicate the candidate miRNA target genes, and lines indicate their interactions. Thus, miRNA-seq analysis showed distinctive miRNA signatures in HULM-EXO compared to UTSM-EXO, suggesting that HULM-EXO might contain a set of miRNAs that are delivered from leiomyomas to healthy endometrium to drive or influence the pathophysiology of uterine leiomyomas. 

### 2.6. Pathway Analysis of Differentially Regulated miRNA Target Genes 

**Figure 6:**
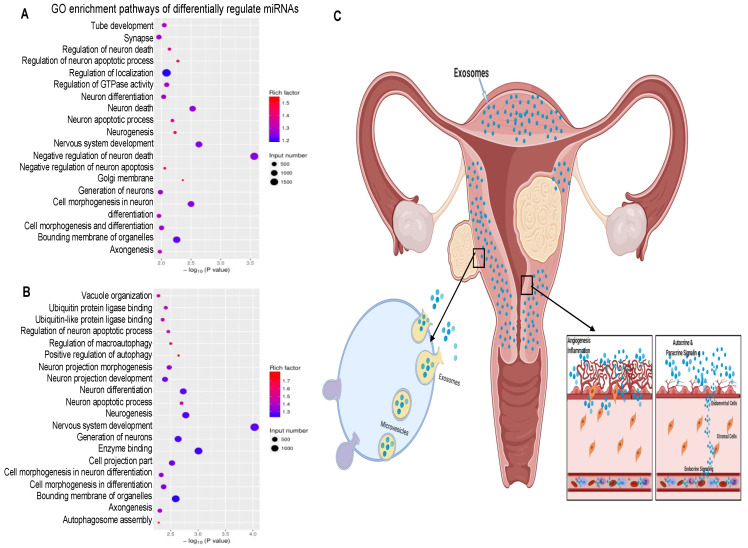
Pathway analysis of differentially regulated miRNA target genes and hypothetical model of human uterine leiomyoma-derived exosome. (**A**,**B**) GO enrichment analysis of differentially miRNA target genes demonstrated that both upregulated and downregulated target miRNAs were significantly enriched in binding membrane organelles, enzyme binding and nervous system development direction in HULM-EXO compared to UTSM-EXO. (**C**) Hypothetical model of human uterine leiomyoma-derived exosome’s action. After they are released, HULM-EXO (HULM-derived exosome) are taken up by neighboring and distant cells and cause abnormal function of the healthy endometrium. The presence of uterine leiomyomas cause alteration in the healthy endometrium and changes in exosome content, leading to increased factors involved in cell proliferation and angiogenesis, among others.

To identify the cellular pathways that are enriched among the differentially regulated miRNA target genes, we performed Gene Ontology (GO) and KEGG enrichment analysis for biological processes and pathways. GO enrichment analysis of genes targeted by the miRNAs downregulated in HULM-EXO compared to UTSM-EXO showed that the most highly enriched pathways were bounding membrane of organelles, regulation of localization, and neuron generation and differentiation, among other biological processes (Figure 6A). GO enrichment analysis of genes targeted by miRNAs upregulated in HULM-EXO compared to UTSM-EXO showed that the most highly enriched categories were enzyme binding, bounding membrane of organelles, and nervous system development, among other biological processes (Figure 6B). Surprisingly, GO enrichment analysis demonstrated that genes targeted by both upregulated and downregulated miRNAs in HULM-EXO compared to UTSM-EXO were enriched in nervous system development, similar to what we have observed in other leiomyoma (tissues and cells) in our laboratory.

## 3. Discussion

Factors expressed and secreted by leiomyomas affect endometrial growth and vessel remodeling, contributing to the increased incidence of reproductive difficulties such as irregular and heavy menstrual bleeding, miscarriages, and subfertility. The objective of this study was to investigate the effect of exosomes derived from human uterine leiomyoma (HULM-EXO) and normal healthy myometrium (UTSM-EXO) cell lines on human endometrium-related endothelial cell line. In the context of leiomyomas, there are no reports about exosome isolation and characterization in leiomyoma tissue, cell lines, or patient-derived exosomes. This study demonstrates the isolation and examination of the functional impact of exosomes from uterine leiomyoma-derived cell line. Several recent studies have demonstrated that exosomes facilitate the transfer of nucleic acids, proteins, and lipids among cells, thereby contributing to intercellular communication [22,23]. Almost all cell types secrete exosomes that are taken up by neighboring and distant recipient cells, suggesting that the tumor microenvironment plays an indispensable role in tumor development and progression [28,29,30,31]. 

In this study, we observed a significant increase in the expression of cell proliferation and angiogenic markers in HEMEC treated with HULM-EXO compared to UTSM-EXO or PBS. There was a slight increase in VEGFA expression in HEMEC treated with UTSM-EXO compared to PBS (negative control), suggesting that UTSM-EXO also established some pro-angiogenic communication with neighboring cells, though not to the extent seen with HULM-EXO. Angiogenesis is indispensable for the establishment and proliferation of tumor development [32,33,34], it is an essential process on menstrual cycle (growth and shedding of the endometrium), and it possibly plays a fundamental role in the irregular and heavy menstrual bleeding observed in women with leiomyomas. Functional examination demonstrated that treatment with HULM-EXO in HEMEC led to increased tube formation, higher total tube length, and increased total branching points compared to UTSM-EXO and PBS control (Figure 4). HULM-EXO may thus alter endometrial angiogenesis, which could contribute to not only increased menstrual bleeding, but disturbed endometrial receptivity and implantation in women with leiomyomas. Together, these findings suggest that HULM-EXO have an adverse influence on the human endometrium, and future studies should investigate the content of the HULM-EXO cargo; this will help to identify mechanistic links to the pathophysiology of uterine leiomyomas.

A hypothetical model of action of human leiomyoma-derived exosomes is presented here. Our proposed model shown in Figure 6C suggests that exosomes secreted by uterine leiomyoma cell line are released into the extracellular space and deliver their cargo to endometrial cells in an autocrine, paracrine, and endocrine manner. After they are released, HULM-EXO are taken up by adjacent endometrial cells and might alter the normal function of the healthy endometrium. The content of exosomes produced by uterine leiomyomas likely enriched in miRNAs might drive cell proliferation and angiogenesis, among other pathologic processes in the endometrium. Alterations in the healthy endometrium in turn change their exosome content, creating a vicious cycle that contributes to irregular and heavy menstrual bleeding in women with uterine leiomyomas, as well as disruption of endometrial receptivity and implantation. 

The transfer of exosome-derived unique miRNAs to recipient cells implicates cell-to-cell communication in leiomyoma pathology [35]. This study identified 71 (48 known and 23 unknown) differentially upregulated miRNAs and 84 (49 known and 35 unknown) downregulated miRNAs in HULM-EXO compared to UTSM-EXO (Figure 5B). Tables S1 and S2 list the known differentially regulated miRNAs (in Supplementary Materials). Importantly, some of the considerably dysregulated miRNAs in HULM-EXO compared to UTSM-EXO have been previously established as functionally relevant in the pathophysiology of leiomyomas. For example, previous studies have found the following miRNAs to be upregulated in uterine leiomyomas tissue or cell lines: miR-21, let-7s, miR-23b, miR-27a, miR-30a, miR-142-3p, miR-142-5p, let-7i-5p, miR-7-5p, miR-483-3p, miR-483-5p, miR-122-5p, miR-494-3p, miR-129-5p, miR-671-3p, miR-181a-5p, miR-335-5p, miR-3180, miR-490-3p, miR-145-5p, miR-34a-5p, miR-136-5p, miR-503-5p, and miR-323b-3p [17,36,37,38,39]. Numerous other miRNAs have been reported as downregulated in uterine leiomyoma tissue or cell lines: miR-29b, miR-32, miR-197, miR-212, miR-126-3p, miR-126-5p, miR-144-3p, miR-144-5p, miR-486-5p, miR-93-5p, miR-29a-3p, miR-29b-3p, miR-143-3p, miR-30a-5p, miR-30b-5p, miR-20c, miR-152-3p, miR-22-3p, miR-654-3p, miR-146a-5p, miR-137, and miR-324-3p [17,36,40,41,42,43]. Furthermore, a comprehensive study performed by Zavadil et al., profiling the expression and function of miRNAs and their relation to target gene expression in uterine leiomyomas demonstrated an inverse association between the most differentially dysregulated miRNA levels and their predicted target gene expression levels. They identified 249 mRNAs that were downregulated and the predicted targets of five upregulated miRNAs (let-7s, miR-21, miR-23b, miR-27a, and miR-30a) while 97 mRNAs were upregulated and the predicted targets of five downregulated miRNAs (miR-29b, miR-32, miR-144, miR-197, and miR-212) in uterine leiomyoma [17]. Correspondingly, some of these miRNAs have been dysregulated in our study. These results indicate that differential pathogenic roles and altered mechanisms are involved in exosomal intercellular communication in the uterine environment. 

Interestingly and as expected, we observed that many miRNAs that are involved in functions related to uterine leiomyoma were differentially regulated in this study, including miR-29b-3p, miR-424-5p, miR-483-3p, and miR-182-5p. These miRNAs are involved in TGFβ signaling, a pathway that plays a key role in the development of leiomyomas [41,44,45,46]. ECM accumulation is another important characteristic of leiomyomas, and we observed that miR-27b-3p, miR-31-5p, miR-101-3p, and miR-29b-1-5p, which have ECM-related functions, were differentially regulated in this study [41,47,48]. miR-130a-3p and miR-222-5p were also differentially expressed; these play a role in angiogenesis and vessel remodeling [49]. miR-155-5p is also involved in inflammation, another common feature of leiomyomas [50,51]. Uterine leiomyomas affect reproductive outcomes, and we observed that miR-223-3p, miR-199b-5p, mir-1244, and miR-183-5p were upregulated in this study, suggesting a potential contribution to the pathology of leiomyomas. Previous studies demonstrated that miR-223-3p played a role in endometrial receptivity by regulating leukemia inhibitory factor (LIF), miR-199b-5p was present in the follicular fluid of women with PCOS and correlated with AMH levels, miR-1244 was upregulated in the follicular fluid of infertile women leading to downstream events that affect fertilization, and miR-183-5p was involved in the regulation of uterine receptivity, enhancement of embryo implantation, and invasion and migration of trophoblast cells [52,53,54,55,56,57,58,59].

One of the limitations of our study was that we used representative HULM and UTSM cell lines to isolate exosomes. Primarily, this work stands as proof-of-concept of important observable differences in the effects of exosomes derived from leiomyoma cell line, and the different miRNA profile between HULM-EXO and UTSM-EXO. Future studies will use fresh primary uterine leiomyomas and healthy myometrium cells isolated from well-matched patients (age, ethnicity, and BMI) to confirm our findings. Another limitation of this study is the lack of proteome profiles in leiomyomas compared to normal myometrium. A comprehensive proteomic analysis of these exosomes will be the topic of future work in our laboratory to complement this study. Overall, this study isolated and portrayed human uterine leiomyoma-derived exosomes, including assessment of the unique miRNA signature compared to exosomes derived from healthy uterine myometrial cells. This investigation also reports on the functional impact of HULM-EXO exosomes on cell proliferation and tube formation (angiogenesis) in human endometrium related cells. Additional studies are being carried out in our lab to confirm and support these findings. Uterine leiomyoma biology is complex, and future studies should aim to establish patient- and patient-derived primary cell exosome functions and signatures. We are currently validating some of the identified dysregulated miRNAs (e.g., miR-223-3p, miR-199b-5p, mir-1244, and miR-183-5p), selected based on their known functions that are closely related to endometrial functions such as receptivity, implantation, and menstrual bleeding. Further studies will address in depth functional studies to uncover their effects in uterine leiomyomas. 

## 4. Materials and Methods

### 4.1. Cell Culture Conditions

Immortalized human uterine leiomyoma (HULM) and immortalized human uterine smooth muscle (UTSM) cell lines were a generous gift from Professor Darlene Dixon, DVM, PhD, National Institute of Environmental Health Sciences, Durham, N.C. [59]. The HULM were obtained from an intramural leiomyoma of a 45-year-old, African American woman; they were immortalized by the introduction of stable human telomere terminal transferase expression (hTERT). The cells were cultured and maintained in phenol red-free, 10% fetal bovine serum, 1% antibiotic solution Dulbecco’s Modified Eagle Medium: Nutrient Mixture F-12. Human endometrial microvascular endothelial cells (HEMEC) from ScienCell research laboratories (# 7010, Carlsbad, CA, USA) were cultured and maintained in ScienCell-formulated endothelial cell medium (ECM) with 5% fetal bovine serum, 1% antibiotic solution, and endothelial cell growth supplement. HEMEC were plated onto fibronectin-coated plates and used for up to six passages to avoid changes in phenotype and gene expression.

### 4.2. Isolation of Exosomes and Treatment

Once HULM and UTSM cells reached 80% confluence, culture media was removed, and cells were washed three times with PBS. Cells were cultured for an additional 48 h with media containing exosome-depleted FBS. The exosomal fraction from the culture media was then isolated using the ExoQuick-TC isolation kit (SBI system Biosciences, Palo Alto, Santa Clara, CA, USA) according to the manufacturer’s recommendations. Briefly, 40 mL cell culture media was centrifuged at 3000× *g* for 15 min at room temperature to remove cells and debris. The supernatant was mixed with 20% of ExoQuick and refrigerated overnight at 4 °C, then the mixture was centrifuged at 1500× *g* for 90 min and the supernatant was removed by aspiration. The pellet was resuspended in 1× PBS and stored at −80 °C or directly processed for experiments. Once HEMEC reached ~80% confluency, they were washed three times with PBS, and then treated with the specified concentrations of exosomes (HULM—UTSM-EXO) or with PBS for 24 or 48 h in FBS-free media.

### 4.3. Characterization of Exosomes from HULM and UTSM Cell Lines

To characterize the particle size distribution of HULM- and UTSM-derived exosomes, nanoparticle tracking analysis (NTA) was performed with measurements taken using a NanoSeries ZetaSizer instrument (Malvern, UK) at the nanotechnology core at the University of Illinois at Chicago. Briefly, 10 μL of exosomes purified from HULM and UTSM culture media were diluted in 1 mL of 1X PBS and disaggregated using a syringe and needle (29-gauge). Then, the sample was injected into the ZetaSizer sample cubicle. The size distribution of exosomes was determined. To visually verify the presence and morphology of HULM- and UTSM-derived exosomes, transmission electron microscopy (TME) was performed. Exosomes were dissolved in PBS, dropped onto a carbon-coated copper grid, and then stained with 2% uranyl acetate. Images of exosomes were acquired using a JEOL 1200EX instrument at the advanced electron microscopy core at the University of Chicago. Immunoblotthing was performed to detect exosome marker proteins and confirm success of isolation. Protein was extracted from HULM- and UTSM-derived exosomes using RIPA lysis buffer with protease and phosphatase inhibitor cocktail (Thermo Scientific, Waltham, MA, USA) according to the manufacturer’s protocol. Protein was quantified by BCA assay (Thermo Scientific). Lysates were cleared by centrifugation and 30 μg of protein was diluted with reducing 4X LDS Sample Buffer (Life Technologies, Carlsbad, CA, USA), resolved on 4–12% Novex Bis-Tris Polyacrylamide Pre-Cast gels (Life Technologies), and transferred onto polyvinylidene difluoride membranes. We probed for exosomal markers CD9 and CD63 (SBI system Biosciences, Palo Alto, Santa Clara, CA, USA). Chemiluminescence Femto HRP reagent (GeneTex, Irvine, CA, USA) was used for detection, and specific protein bands were visualized using ChemiDoc XRS molecular imager (Bio-Rad, Hercules, CA, USA). 

### 4.4. Exosome Labeling

75 μg of HULM- and UTSM-derived exosomes were labeled using the ExoGlow-Membrane EV labeling kit (SBI System Biosciences) and incubated for 30 min at RT according to the manufacturer’s protocol. Then, the labeled reaction was centrifuged at 10,000 rpm for 10 min, the supernatant was carefully aspirated, and the labeled exosomes pellet was resuspended in PBS and added to HEMEC. The uptake of labeled exosomes by HEMEC was detected by a Leica TCS SP5 II laser scanning confocal microscope.

### 4.5. Cell Proliferation Assay

A bromodeoxyuridine (BrdU)-incorporation assay (Abcam) was used to measure cell proliferation after treatment of HEMEC with HULM-derived exosomes, UTSM-derived exosomes, or PBS as a control. A total of 1 × 10^5^ cells per well were plated in a 96-well plate, then treated with exosomes or PBS for 48 h. BrdU incorporation was allowed to proceed for 4 h at 37 °C, followed by DNA denaturation, BrdU antibody labeling, and detection at 492 nm/370 nm absorbance using the Synergy HT plate reader (BioTek Instruments Inc., Winooski, VT, USA). BrdU incorporation was then quantified to compare cells treated with HULM-derived exosomes versus UTSM-derived exosomes, using PBS as a negative control; the final absorbance was calculated by subtracting the absorbance at 492 nm from the absorbance at 370 nm. The assay was performed four times with triplicate samples each run. 

### 4.6. RNA Extraction and Gene Expression

RNA from exosome (HULM- UTSM-EXO) treated and untreated HEMEC was extracted according to RNeasy kit (QIAGEN, Valencia, CA, USA). The concentration of total RNA was determined using NanoDrop (Thermo Scientific). 1 μg of cDNA was synthesized using RNA to cDNA EcoDry (Takara, Mountain View, CA, USA). Real-time quantitative PCR (qPCR) was performed on a CFX96 PCR instrument using SYBR Green Supermix (Bio-Rad) and primers to amplify C-MYC, VEGFA, and BCL2 genes and 18S and β-ACTIN as housekeeping genes. The results are presented as relative gene expression using CFX Maestro™ software. This assay was performed three times with triplicate samples in each experiment.

### 4.7. Endothelial Tube Formation Assay

Once HEMEC reached ~80% confluency, cells were harvested, resuspended in FBS-free media, and seeded at a density of 120,000 cells/well in growth-factor-reduced Matrigel Basement Membrane Matrix (Corning). Immediately, HEMEC were treated with 30 μg total of HULM- or UTSM-derived exosomes or PBS on a 24-well plate (BD Bioscience) and incubated up to 18 h at 37 °C with 5% CO_2_. Tube formation was examined under an inverted microscope and imaged at 4× magnification; the whole well was analyzed divided in four selected regions. Total tube length and branching points were measured using ImageJ software, and these are representative images of multiple independent experiments. Results are shown as the mean and standard errors, and the experiment was run three times with triplicate samples in each experiment. 

### 4.8. miRNA Extraction, Small RNA-Sequencing, and Bioinformatic Analysis

miRNA was extracted from exosomes using the SeraMir Exosome RNA column purification kit (SBI System Biosciences) according to the manufacturer’s protocol at Creative Biolabs biotech company. Agarose gel electrophoresis was used to analyze the degree of RNA degradation or contamination, nanodrop (Thermo Fisher Scientific) was used to detect the purity of RNA (OD 260/280), Qubit was used for accurate quantification of RNA concentration, and Agilent 2100 was used to accurately detect the integrity of RNA. Library preparation: the library was constructed by using Small RNA Sample Prep kit. Using the special structure of 3′ and 5′ end of small RNA, the cDNA was directly synthesized by splicing the ends of small RNA with total RNA as the starting sample. After PCR amplification, the target DNA fragment was separated by PAGE gel electrophoresis, and the cDNA library was obtained by gel cutting. After the construction of the library, Qubit2.0 was used for preliminary quantification, and the library was diluted to 1ng/μL, and then the insert size of the library was detected by Agilent 2100. After the insert size was obtained, the effective concentration of the library was accurately determined by qPCR (>2nM) to ensure the quality of the library. Sequencing: the different libraries were pooled according to the effective concentration and the demand of target offline data, and then the HiSeq/MiSeq sequencing was performed. Data analysis: the original sequenced data obtained from illumine HiSeq was transformed into sequence data through base calling FASTQ format, and the original sequencing data file was obtained. FASTQ format file can record the base number and quality fraction of the read. FASTQ format was stored in the unit of sequenced reading segment, each reading segment occupies 4 lines, of which the first line and the third line are composed of sequence identifiers and read segment name. The second line is the base sequence, and the fourth line is the quality fraction of sequencing data of the base at the corresponding position. Sequence alignment on reference genome, using bowtie to locate the screened sRNA on the reference sequence, and analyze the distribution of sRNA on the reference sequence. Align the reads mapped on the reference sequence with the sequence in the specified range in miRbase to obtain the detailed information of sRNA on each sample, including the secondary structure of known miRNA on the matching, the sequence, length, and occurrence times of miRNA in each sample. Mirdeep was used to predict novel miRNAs, the data containing known miRNAs was filtered out, the data containing non-miRNA sRNA was annotated to filter out ncRNA, repeat, and other partial data, and the rest of the reads were used to predict novel miRNAs. miRNA target gene was predicted using miRanda and RNAhybrid software. Finally, examination of the uterine leiomyoma literature was performed to identify the possible role of the newly identified miRNAs in outcomes related to leiomyoma pathogenesis. PubMed and Google Scholar searches were used to identify relevant articles using the following keywords either alone or in combination with uterine fibroid(s), uterine leiomyoma, and the respective miRNA.

### 4.9. Statistical Analysis

Each set of experiments was repeated at least three times, followed by statistical analysis. All the data were presented as the mean ± SD. *p* values were analyzed using Student’s *t* test or two-way ANOVA analysis with Tukey multiple comparison post-test employing GraphPad version 7 (GraphPad Software Inc., San Diego, CA, USA). Values were considered statistically significant when *p* < 0.05.

## 5. Conclusions 

Our findings are consistent with extensive literature describing the role of exosomes in modifying disease pathophysiology and emphasize the importance of investigating the exosomal signatures of primary uterine leiomyoma cells as possible biomarkers or therapeutic targets. Currently, there are no effective long-term treatments to improve severe leiomyomas symptoms. Unfortunately, surgery is still the main stay for severe cases, which compromises the fertility potential of affected patients, especially in women of color who suffer early onset of high burden disease. The discovery of extracellular vesicles containing cargo with a unique miRNA signature opens potential applications as diagnostic markers or therapeutic vehicles. Some of the identified miRNAs in this study might be suitable candidates and/or targets for novel non-surgical treatment options for women with uterine leiomyomas.

## Data Availability

Data is contained within the article and supplementary material.

## Supplementary Materials

The following supporting information can be downloaded at: https://www.mdpi.com/article/10.3390/ph15050577/s1, Table S1: List of differentially downregulated miRNAs in HULM-EXO compared to UTSM-EXO; Table S2: List of differentially upregulated miRNAs in HULM-EXO compared to UTSM-EXO.
