# Supplementary material for: Report of Exosomes Isolated from a Human Uterine Leiomyoma Cell Line and Their Impact on Endometrial Vascular Endothelial Cells
Source: Pharmaceuticals (Basel). 2022 May 5;15(5):577. doi: 10.3390/ph15050577 (PMC9143402; doi:10.3390/ph15050577)
Supplement: Supplementary file 1 [file pharmaceuticals-15-00577-s001.zip › pharmaceuticals-1643036-supplementary.pdf]

Table S1. List of differentially downregulated miRNAs in HULM-EXO compared to UTSM-EXO

miRNA	Log FC	P value	FDR
hsa-miR-152-3p	-0.6171983	0.044064243	0.14976265
has-miR-30c-5p	-0.6244887	0.018638442	0.07200607
hsa-miR-29a-3p	-0.6812954	0.001393438	0.00779454
hsa-miR-181a-5p	-0.8437619	0.003043485	0.01527431
hsa-miR-27b-3p	-0.8550467	0.000191049	0.00136791
hsa-miR-941	-0.8669564	0.014002782	0.05762
hsa-miR-224-5p	-0.8907374	0.001273641	0.00719942
hsa-miR-744-5p	-0.9034923	0.025964196	0.09420793
hsa-miR-654-3p	-0.9044126	0.023508844	0.0876684
hsa-miR-27a-3p	-0.9622595	8.22E-05	0.00060453
hsa-miR-24-3p	-1.0148283	8.06E-06	7.21E-05
hsa-miR-29b-3p	-1.0271054	1.23E-06	1.27E-05
hsa-miR-28-3p	-1.0505567	0.005047699	0.02357056
hsa-miR-23a-3p	-1.1126003	3.44E-07	3.70E-06
hsa-miR-130a-3p	-1.1357986	0.008781508	0.03833878
hsa-miR-23b-3p	-1.237715	1.07E-05	9.40E-05
hsa-miR-320c	-1.3507584	0.000262323	0.00182945
hsa-miR-7-5p	-1.4017724	1.54E-07	1.76E-06
hsa-miR-30a-5p	-1.4310428	5.91E-08	7.25E-07
hsa-miR-181a-2-3p	-1.4756243	0.007180229	0.03240154
hsa-miR-320b	-1.4973795	4.80E-05	0.00039641
hsa-miR-143-3p	-1.5006143	1.09E-11	1.78E-10
hsa-miR-22-3p	-1.5243372	4.87E-07	5.13E-06
hsa-miR-181b-5p	-1.5917024	1.27E-06	1.29E-05
hsa-miR-197-3p	-1.5929096	0.005823769	0.02672961
hsa-miR-136-5p	-1.5984208	0.000418716	0.00270904
hsa-miR-190a-5p	-1.7061308	0.001255385	0.00719942
hsa-miR-503-5p	-1.7408825	0.001847602	0.0101241
hsa-miR-1296-5p	-1.7554006	0.010113281	0.04276246
hsa-miR-143-5p	-2.0170247	0.001822828	0.01009133
hsa-miR-6087	-2.0435692	5.94E-08	7.25E-07
hsa-miR-424-5p	-2.3900127	3.37E-10	4.90E-09
hsa-miR-7704	-2.542874	0.016084066	0.06543291
hsa-miR-146a-5p	-2.5996335	1.37E-05	0.0001184
hsa-miR-145-5p	-2.6264697	2.50E-17	5.16E-16
hsa-miR-660-5p	-2.7726722	0.013951236	0.05762
hsa-miR-320d	-2.8440097	2.33E-07	2.55E-06
hsa-miR-323b-3p	-2.940843	0.007880255	0.03468604
hsa-miR-34a-5p	-3.147216	1.31E-25	4.39E-24
hsa-miR-324-3p	-3.9874603	0.000667207	0.00407148
hsa-miR-4508	-4.2397756	3.60E-18	7.74E-17
hsa-miR-335-5p	-4.5554876	1.35E-24	4.03E-23
hsa-miR-137	-4.6679459	2.46E-10	3.67E-09
hsa-miR-3180	-5.8461076	0.03361661	0.11722156
hsa-miR-3180-3p	-5.8461076	0.03361661	0.11722156
hsa-miR-335-3p	-6.833513	0.000667207	0.00407148
hsa-miR-490-5p	-6.969866	0.000352487	0.00230836
hsa-miR-490-3p	-7.2091024	5.33E-05	0.0004207
8hsa-miR-3180-3p	-5.8461076	0.03361661	0.11722156

Table S2. List of differentially upregulated miRNAs in HULM-EXO compared to UTSM-EXO

miRNA	Log FC	P value	FDR
hsa-miR-23a-5p	5.825600757	0.017297867	0.067311266
hsa-miR-1244	5.658852087	0.03361661	0.117221556
hsa-miR-6724-5p	5.658852087	0.03361661	0.117221556
hsa-miR-150-5p	3.342380532	0.002009464	0.010790821
hsa-miR-483-5p	3.340968643	9.33E-10	1.28E-08
hsa-miR-96-5p	2.23550084	0.001905282	0.010334711
hsa-miR-222-5p	2.157393072	0.010113281	0.042762455
hsa-miR-25-5p	2.151013595	0.004181329	0.020412488
hsa-miR-106b-5p	2.083186416	0.041975388	0.14357187
hsa-miR-671-3p	1.991038057	0.041975388	0.14357187
hsa-miR-483-3p	1.732686365	0.00108952	0.006429366
hsa-miR-301a-3p	1.688598524	0.000282516	0.001945013
hsa-miR-142-3p	1.641688378	1.99E-14	3.82E-13
hsa-miR-126-3p	1.610200192	8.64E-13	1.60E-11
hsa-miR-29b-1-5p	1.599557063	0.034844927	0.120720812
hsa-miR-486-5p	1.573487885	1.33E-12	2.39E-11
hsa-miR-122-5p	1.565230091	1.80E-11	2.85E-10
hsa-miR-129-5p	1.555694143	0.002593418	0.013263481
hsa-miR-182-5p	1.461648808	6.78E-05	0.00051268
hsa-miR-183-5p	1.441585693	0.023844974	0.087703774
hsa-miR-17-5p	1.441585693	0.023844974	0.087703774
hsa-miR-142-5p	1.393506727	2.19E-08	2.80E-07
hsa-miR-223-3p	1.328928957	0.000807467	0.004817886
hsa-miR-1306-5p	1.285320937	0.004406531	0.021318082
hsa-let-7i-5p	1.253668609	3.66E-09	4.79E-08
hsa-miR-101-3p	1.242125979	1.37E-07	1.60E-06
hsa-miR-18a-5p	1.228171743	0.02594261	0.094207926
hsa-miR-144-5p	1.225458383	4.08E-05	0.000342382
hsa-miR-199b-5p	1.2051242	0.00398251	0.019620256
hsa-miR-93-5p	1.193607476	8.15E-08	9.73E-07
hsa-miR-151a-5p	1.188763663	0.02294735	0.086779768
hsa-miR-494-3p	1.157946784	0.002989694	0.015145902
hsa-miR-155-5p	1.150942157	4.55E-06	4.21E-05
hsa-miR-126-5p	1.099380132	3.10E-05	0.000264508
hsa-miR-16-5p	1.087643177	1.66E-07	1.86E-06
hsa-miR-92a-3p	1.008295903	5.98E-06	5.44E-05
hsa-miR-148a-3p	1.007272756	0.002265509	0.012045332
hsa-miR-487b-3p	0.963198953	0.047810735	0.160464778
hsa-miR-25-3p	0.866205963	6.62E-05	0.000507991
hsa-miR-144-3p	0.844947407	0.00023011	0.001625908
hsa-miR-382-5p	0.833937717	0.003216573	0.015993515

hsa-miR-194-5p	0.81067085	0.045967706	0.155249422
hsa-miR-451a	0.775002044	0.000608198	0.003888126
hsa-miR-432-5p	0.723622781	0.016613321	0.067077846
hsa-miR-379-5p	0.678468518	0.020651183	0.079212037
hsa-miR-20a-5p	0.665026875	0.023269266	0.08738179
hsa-let-7g-5p	0.633042044	0.007334363	0.032821275
hsa-miR-423-5p	0.629785733	0.005515075	0.025530993
hsa-miR-31-5p	0.602595019	0.007644987	0.033928581
